# Increased daily physical activity and fatigue symptoms in chronic fatigue syndrome

**DOI:** 10.1186/1476-5918-4-3

**Published:** 2005-03-03

**Authors:** Christopher D Black, Patrick J O'Connor, Kevin K McCully

**Affiliations:** 1 Department of Exercise Science, The University of Georgia, Athens, GA, USA

**Keywords:** Mood, Pain, Exercise, Vigor, Fibromyalgia, Accelerometry

## Abstract

Individuals with chronic fatigue syndrome (CFS) have been shown to have reduced activity levels associated with heightened feelings of fatigue. Previous research has demonstrated that exercise training has beneficial effects on fatigue-related symptoms in individuals with CFS.

**Purpose:**

The aim of this study was to sustain an increase in daily physical activity in CFS patients for 4 weeks and assess the effects on fatigue, muscle pain and overall mood.

**Methods:**

Six CFS and seven sedentary controls were studied. Daily activity was assessed by a CSA accelerometer. Following a two week baseline period, CFS subjects were asked to increase their daily physical activity by 30% over baseline by walking a prescribed amount each day for a period of four weeks. Fatigue, muscle pain and overall mood were reported daily using a 0 to 100 visual analog scale and weekly using the Profile of Mood States (Bipolar) questionnaire.

**Results:**

CFS patients had significantly lower daily activity counts than controls (162.5 ± 51.7 × 10^3 ^counts/day vs. 267.2 ± 79.5 × 10^3 ^counts/day) during a 2-week baseline period. At baseline, the CFS patients reported significantly (P < 0.01) higher fatigue and muscle pain intensity compared to controls but the groups did not differ in overall mood. CFS subjects increased their daily activity by 28 ± 19.7% over a 4 week period. Overall mood and muscle pain worsened in the CFS patients with increased activity.

**Conclusion:**

CFS patients were able to increase their daily physical activity for a period of four weeks. In contrast to previous studies fatigue, muscle pain, and overall mood did not improve with increased activity. Increased activity was not presented as a treatment which may account for the differential findings between this and previous studies. The results suggest that a daily "activity limit" may exist in this population. Future studies on the impact of physical activity on the symptoms of CFS patients are needed.

## Introduction

Chronic fatigue syndrome (CFS) is characterized by persistent debilitating fatigue often accompanied by a complex of other symptoms (e.g., impaired memory, sore throat, post-exertional fatigue, aching and stiffness in muscles) lasting at least six months that is unresolved with rest or medication [1,2]. A primary component of the case definition of CFS is the inability of patients to maintain their own pre-illness level of activity. Using both self-report as well as accelerometers, previous research on activity levels in this population suggests that individuals with CFS have physical activity levels that are 15% to 40% reduced from those of otherwise healthy sedentary individuals [3-6]. These data suggest that most CFS patients are on the lowest end of the activity spectrum.

It is widely accepted that a sedentary lifestyle may greatly increase the risk of development of cardiovascular disease and Type II diabetes, as well as contributing to bone loss, and an age-related loss of function in a person's ability to perform daily activities [7-9]. Recent recommendations by the Surgeon General suggest that accumulating thirty minutes of moderate intensity physical activity per day could provide positive health benefits [10]. Individuals are encouraged to increase their daily physical activity not only by traditional, structured exercise programs, but also by increasing the amount of unstructured physical activity they perform each day (i.e. the amount they walk each day). The CFS population, with its low daily activity, could derive many positive health benefits from increasing its daily activity, and given their extremely sedentary nature the needed exercise stimulus could easily be met by increasing the amount of walking each subject performs daily.

Few randomized controlled trials designed to assess the efficacy of exercise training have been conducted in the CFS population. Two studies used graded exercise performed several days-a-week over a period of 12–26 weeks and both reported an improvement in fatigue-related symptoms and aerobic capacity [11,12]. Additionally, a single subject performed graded aerobic exercise as well as strength training and reported moderate improvements in fatigue-related and other CFS symptoms [13]. Results from these studies suggest that exercise, in addition to providing positive health outcomes, could also provide beneficial effects to CFS symptomology. Although exercise has been shown to be beneficial, it is unknown to what extent a structured, formal exercise program may alter daily activity in CFS patients. It is common for CFS patients to report exacerbation of their symptoms when too much physical activity is undertaken. CFS patients may have to "rest" more often and/or decrease other types of physical activity in order to compensate for their periods of exercise.

The purpose of this study was to assess normal daily physical activity levels in CFS patients using accelerometers over a two-week period and compare these values to a group of healthy sedentary individuals. Additionally, the CFS patients were asked to increase their daily physical activity approximately 30% over a four week period by walking a prescribed amount each day while maintaining their non-exercise daily physical activity. Daily ratings of mood, fatigue, and muscle pain intensity were assessed and compared between the groups as well as in response to increased daily activity in the CFS patients.

## Methods

### Participants

All experimental procedures were approved by the Institutional Review Board at the University of Georgia, and informed consent was obtained from each participant. All participants were recruited from the general community and either responded to a newspaper ad, responded to a flyer placed around campus, or were referred to the study by their physician. Seventeen CFS and twenty-one controls responded and were screened as possible study participants. A physician's diagnosis of CFS was required for inclusion. Additionally, CFS patients were required to confirm a self-report of decreased physical activity compared to pre-CFS levels, and self-reported inability to sustain high levels of physical activity without a subsequent exacerbation of CFS symptoms for study inclusion. CFS participants with a self-reported history of depression or other psychiatric illness were excluded. Control participants were chosen to be similar in age, height, and weight to the CFS patients and were defined as sedentary by self-report of one bout of regular exercise per week or less. The most sedentary participants (those with desk jobs, etc.) were given first priority for study inclusion. Control participants were also apparently healthy and reported no illnesses or disease conditions. Medications were monitored in all participants. The CFS participants were found to be taking many medications, both prescription and over-the-counter. Analgesics such as Vioxx, Celebrex, Advil, and Aleve were common.

### Study Design

Initially, participants received instructions for wearing the activity monitors and for completing a daily activity log. A "pre" score from the 30-item Profile of Mood States short form questionnaire (POMS) was obtained (The Educational and Industrial Testing Service, San Diego, CA). Participants proceeded to wear the monitors for two weeks during which time they were instructed to maintain normal daily activity. After the two weeks, data were collected from the activity monitors, and the monitors were reset. The participants then wore the monitors for an additional four weeks. The CFS patients were asked to increase their daily physical activity (30% above baseline) during this four week period by walking a prescribed amount each day in order to approximate the daily physical activity of a healthy sedentary person. This was based upon averaging the findings of others that suggested CFS patients had activity levels that were 15% to 40% reduced from healthy but sedentary individuals [3,4,6]. CFS patients were given neutral instructions as to whether or not increasing their daily physical activity would alter their mood and fatigue symptoms. Control participants maintained their normal activity for a six week period. Daily activity logs were completed each day. Participants recorded all daily activities, time spent in each activity, as well as time periods when the monitor was not worn (e.g., bathing). Participants also completed a series of questions documenting their daily mood, perceptions of fatigue and muscle pain intensity, and the duration of time fatigue and muscle pain were experienced each day.

### Objective Measurement of Physical Activity

Daily physical activity was assessed by a CSA accelerometer (Computer Science Associates Inc., Fort Walton Beach, FL). To ensure accurate measurements, the procedure recommended by CSA was followed – the monitors were positioned over the subject's anterior superior iliac spine with the belt fitting snuggly so as to limit extraneous monitor movement. Participants were asked to wear the monitors at all times of the day, including sleep. Two minute epochs were used. Data were retrieved from the monitors using a specially designed docking module that input data into a computer.

Recommended percent increases in daily activity were calculated based on each subject's average daily counts during their two-week baseline activity period. Counts are arbitrary units assigned to movements detected by the accelerometer. Counts are assigned based upon the magnitude of a change in velocity during a given time period. The number of counts needed to raise daily activity approximately 30% was calculated. Participants were then asked to walk on a treadmill at what they considered to be a comfortable walking pace. Walking speed was recorded, and used to calculate the recommended daily walking time. The approximate number of counts per minute for various walking speeds was assessed prior to the onset of the study (unpublished observations). Additionally, a pedometer was also used to aid participants in achieving the desired daily activity increase. Participants were given an approximate number of steps to take each day during their walk based upon their prescribed walking pace and time. Steps per minute for various walking speeds were assessed in a similar manner as counts per minute prior to the study (unpublished observations).

### Self-report of Daily Activity and Feelings

Data concerning daily activity, mood, fatigue, and muscle pain were obtained from each subject via daily self-report. Participants were asked to report all daily activities and time spent engaged in each. Time periods when the activity monitor was not worn were also reported. A series of five questions concerning daily mood, fatigue intensity, and muscle pain intensity were also answered. Participants ranked, using a 10 cm (0 to 100 mm) visual analog scale their general overall daily mood for that day (with 0 being their best possible overall mood and 100 being their worst possible overall mood), the intensity of their fatigue that day (with 0 being no fatigue and 100 being the highest intensity fatigue imaginable), and the intensity of their muscle pain that day (with 0 being no pain and 100 being the worst imaginable pain). A similar visual analog scale has been used previously in CFS patients to rate daily fatigue [13]. Additionally, participants reported the amount of time each day they experienced fatigue as well as muscle pain. Once each week participants completed a Profile of Mood States (POMS) short form questionnaire consisting of 30 questions (The Educational and Industrial Testing Service, San Diego, CA) in which participants reported how they had been feeling during the prior seven days. These forms were scored for both fatigue and vigor ratings.

### Statistical analysis

Independent samples t-tests were conducted to compare for differences in subject characteristics between the groups. A repeated measures ANOVA was conducted to determine differences between activity level and mood in CFS and control participants. When a significant interaction was observed a one-way repeated measure ANOVA was performed to analyze simple effects with planned comparisons performed to analyze differences in treatment means. All values are reported means ± SD. Analyses were conducted and significance was assumed at an α level of 0.05.

## Results

### Participants

No participants (CFS or controls) reported adverse events associated with the increased activity program. Data were obtained from six CFS patients as well as seven sedentary control participants. Additionally, two other CFS patients began the testing protocol but were removed from the study at their own request. One was removed on doctor's recommendation due to an injury (unrelated to the study) and the second was removed due to a change in residence. The physical characteristics of all participants are presented in Table 1. Mean age, height, and weight, were not different between the CFS patients and the sedentary controls. Five of the six CFS patients also had a physician's diagnosis of fibromyalgia.

**Table 1:** Participants characteristics for six CFS and seven sedentary control participants, values are mean ± SD.

	**Age (Years)**	**Height (cm)**	**Weight (kg)**
**CFS**	43 ± 4.6	164 ± 7.3	73 ± 21.2
**Control**	43 ± 6.5	167 ± 7.0	70 ± 16.7

### Daily Physical Activity

Individual as well as mean group daily activity counts are presented in Table 2. All 24-hour periods in which the monitor was not worn for at least 23 hours were removed from the analysis. No trends were observed in daily activity counts in either subject group across all monitoring periods. Day-to-day counts were also relatively stable within a given activity period. Based on this, activity levels are presented as average daily counts during a given activity period. During baseline activity, CFS participants demonstrated 39% lower daily activity counts compared to controls (*P *= 0.017). All six of the CFS participants were successful in increasing their daily physical activity. Their daily activity counts increased 28%, on average, during the four-week training period (*P *< 0.001). However, it should be noted that 4 of the 6 CFS participants did not reach the prescribed 30% increase in daily activity. Interestingly, following their activity increase the CFS participants had activity levels that were still 24% reduced from those of the control group (*P *= 0.08).

**Table 2:** Average daily activity counts for CFS and control participants. Data are mean ± SD.

**Subject**	**Average Daily Activity **(Coun'ts × 10^3^)		**%Difference**
**CFS**	**Baseline**	**Increase**	

1	88.3 ± 24.1	143.4 ± 42.5	+ 62.3
2	126.7 ± 19.5	179.1 ± 44.9	+ 41.4
3	199.8 ± 38.7	226.7 ± 57.5	+ 13.5
4	167.4 ± 32.8	197.4 ± 32.5	+ 17.9
5	234.2 ± 37.2	263.8 ± 31.2	+ 12.6
6	158.3 ± 32.9	193.5 ± 33.0	+ 22.2
**Mean**	**162.5 ± 51.7**	**200.6 ± 41.2**	

**Control**	**Baseline 1**	**Baseline 2**	

1	415.1 ± 79.4	360.7 ± 67.3	- 13.1
2	284.1 ± 87.1	289.5 ± 81.3	+ 1.9
3	150.6 ± 61.6	146.2 ± 48.7	- 3.0
4	254.9 ± 60.8	281.9 ± 76.5	+ 10.6
5	223.7 ± 73.5	190.9 ± 68.8	- 14.7
6	263.7 ± 60.7	233.9 ± 58.1	- 11.3
7	278.1 ± 106.2	274.0 ± 113.9	- 1.5
**Mean**	**267.2 ± 79.5**	**253.9 ± 70.0**	

### Self-Report Mood/Feeling Ratings

**Figure 1:**
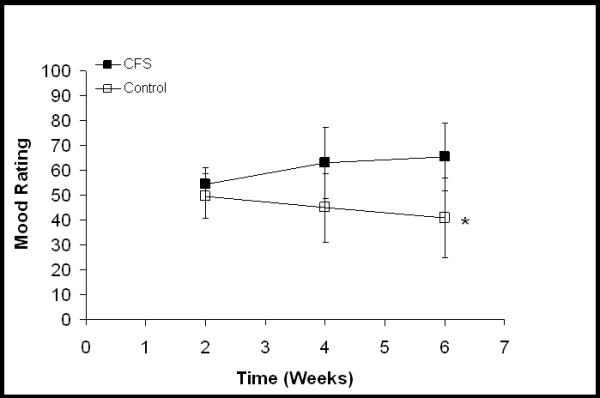
Overall mood ratings. Each time point represents an average of the scores from the previous two weeks. "0" represents the best possible mood and "100" represents the worst mood imaginable. For CFS participants, the two week time point is from baseline activity and the four and six week time points are from increased activity. For control participants, all time points are from baseline activity. * Significant group × time interaction (*P *= 0.016). Values are mean ± SD.

**Figure 2:**
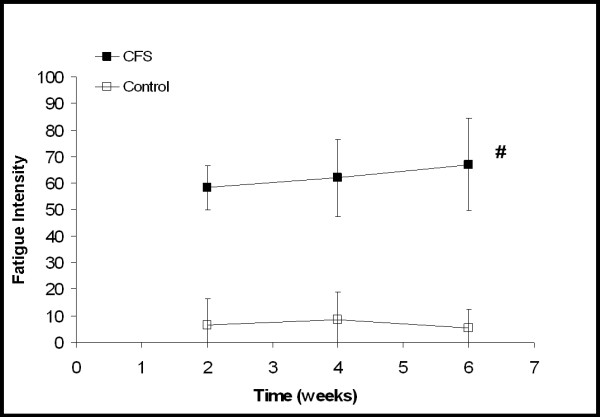
Ratings of fatigue intensity with "0" being a complete lack of fatigue and "100" being the highest intensity fatigue imaginable. Each time point represents an average of the daily scores from the previous two weeks. For CFS participants, the two week time point is from two weeks of baseline activity and the four and six week time points are from increased activity. For the control participants all time points are from baseline activity. CFS participants did not change significantly over time with increased activity. # Significant difference between CFS and control participants (*P *< 0.001). Values are mean ± SD.

**Figure 3:**
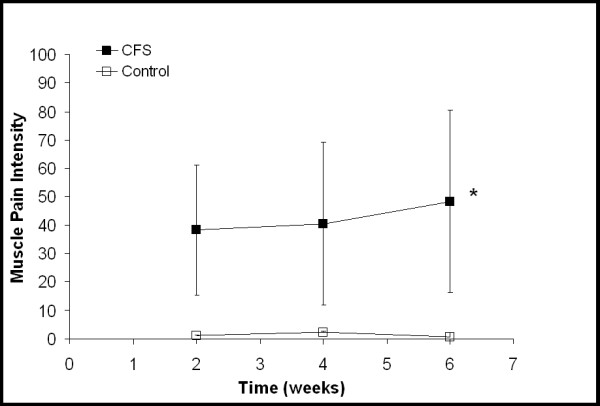
Ratings of muscle pain intensity with "0" being a complete lack of muscle pain and "100" being the worst muscle pain imaginable. Each time point represents an average of the daily scores from the previous two weeks. For CFS participants, the two week time point is from two weeks of baseline activity and the four and six week time points are from increased activity. For the control participants all time points are from baseline activity. * Significant group by time interaction (*P *= 0.030). Values are mean ± SD.

Figures 1, 2, and 3 contain daily ratings of overall mood, fatigue intensity, and muscle pain intensity averaged over two week periods. Days where missing data were found (i.e. ratings were not completed), were checked against the activity monitor data to ascertain if the participants simply forgot to fill out the form or if some problem was present that could prevent them from filling out the ratings. Out of 692 possible days, missing data were found for only 19 days. Activity monitor data appeared normal on all 19 of these days. This suggests that the missing data were likely the consequence of participants forgetting to fill out the form rather than due to any adverse medical event.

Figure 1 demonstrates a significant group-by-time interaction between the CFS and control participants (*P *= 0.016, Eta^2 ^= 0.311) in overall mood. CFS participants reported a worsening of overall mood over time compared to controls. Neither an interaction nor a time main effect was observed in ratings of fatigue intensity. A significant group difference was observed between the CFS and control participants (Fig. 2, *P *< 0.001, Eta^2 ^= 0.892). Although not statistically significant, ratings of fatigue intensity in the CFS group did increase from 58.2 ± 8.5 to 67.0 ± 17.5 (indicating a worsening of symptoms). A significant group-by-time interaction (*P *= 0.03, Eta^2 ^= 0.295) was seen between the CFS and control participants in their ratings of muscle pain intensity (Fig. 3). As their daily activity was increased, the CFS participants reported higher intensity muscle pain compared to controls.

The amount of time spent with fatigue each day as well as the amount of time spent with muscle pain each day was also reported by both groups. During baseline activity, the CFS participants reported experiencing a significantly greater amount of time spent with fatigue per day compared to the control participants (930 ± 397 min/day. vs. 43 ± 73 min/day; *P *< 0.001). Additionally, the CFS participants also reported experiencing a significantly greater amount of time spent with muscle pain each day (552 ± 505 min./day vs. 9 ± 22 min./day; *P *= 0.011). A significant time main effect was found for time spent with fatigue each day (*P *= 0.047, Eta^2 ^= 0.243). A significant difference was found between baseline activity, 451 ± 528 min/day, compared to the final two weeks of increased activity 521 ± 566 min/day (*P *= 0.048, Eta^2 ^= 0.287). The CFS participants also demonstrated a non-significant increase in time spent with muscle pain each day during baseline activity, the first two weeks of increased activity and the final two weeks of increased activity (554 ± 507 min/day vs. 642 ± 546 min/day vs. 713 ± 557 min/day). Control participants demonstrated no change over time in time spent each day with fatigue or muscle pain.

**Figure 4:**
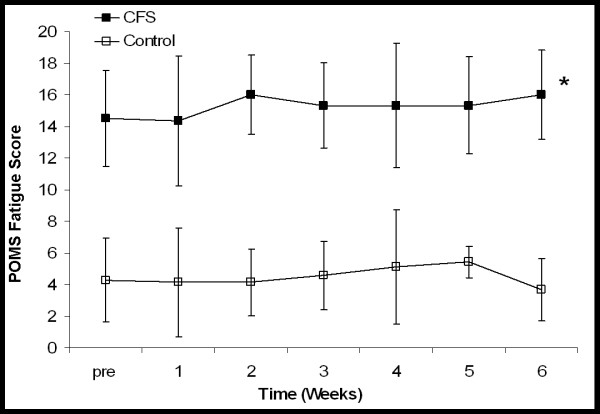
Mean POMS short form ratings (taken once a week) scored for fatigue. Higher scores represent greater fatigue. For CFS participants scores were obtained prior to beginning the study (pre), at the end of each week of baseline activity (1–2) and after each of four weeks of increased activity (3–6). For control participants scores were obtained prior to beginning the study and at the end of each of six weeks of baseline activity (1–6). * Significant difference between the CFS and control participants at all time points (*P *< 0.001). Scores did not change over time in either group. Values are mean ± SD.

**Figure 5:**
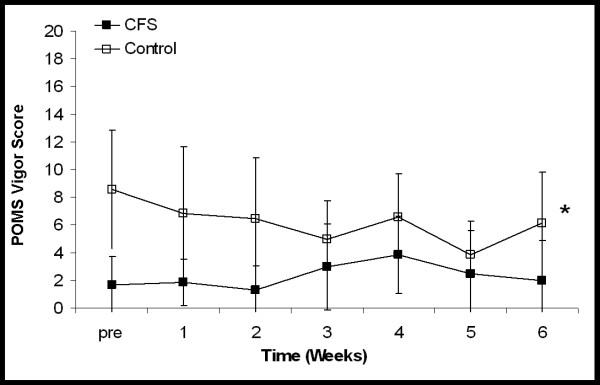
Mean POMS short form ratings (taken once a week) scored for vigor. Higher scores represent greater vigor. For CFS participants scores were obtained prior to beginning the study (pre), at the end of each week of baseline activity (1–2) and after each of four weeks of increased activity (3–6). For control participants scores were obtained prior to beginning the study and at the end of each of six weeks of baseline activity (1–6). * Significant difference between CFS and control participants (*P *< 0.036). Values are mean ± SD.

Figures 4,5 shows the mean weekly scores on the POMS fatigue and vigor scale for the CFS and control participants. A large and significant difference was observed between the CFS participants and the control participants with respect to their fatigue scores (*P *< 0.001, Eta^2 ^= 0.916). No change was observed in the fatigue scores of the CFS participants as their daily physical activity was increased. The control participants also demonstrated no change over time. Similarly, a significant difference was also observed between the CFS and control groups when vigor scores were compared (*P *= 0.036, Eta^2 ^= 0.343). No change was observed in the vigor scores over time or in response to increased activity in either group.

## Discussion

A primary finding of this study was that individuals with CFS can increase their daily physical activity 28% on average over a four week period. Average daily activity counts, measured by accelerometer, increased in all six of the CFS participants who participated in the study. The magnitude of the observed increases in daily activity ranged from approximately 13% to 60%. These results are consistent with previous studies that have shown that CFS patients can sustain training programs lasting 12 to 26 weeks [11,12]. The major difference between this study and previous training studies was that our goal was to increase total daily physical activity opposed to participating in an aerobic training program several days per week. Participation in a traditional program, even if the subject is compliant, does not insure that the subject increased total daily physical activity. With a traditional training program, it is conceivable that the CFS patients could "rest" between training sessions and consequently not experience a net gain in daily activity. This study demonstrates that with encouragement CFS patients can not only exercise daily, but also sustain enough of their non-exercise daily activities to result in sustained increases in daily physical activity over a period of four weeks.

Another interesting aspect of this study was the fact that even after increasing their daily activity 28%, our CFS participants were still approximately 25% less active than our sedentary control participants. We attempted a 30% increase in daily physical activity with the hope of bringing the daily activity levels of our CFS participants up to those of sedentary individuals. This was based upon the findings of others that suggested CFS patients had activity levels that were 15% to 40% reduced from healthy but sedentary individuals [3,4,6]. Our results suggest that previous estimates of daily activity in this population may have been overestimated. However, given our small sample size it is possible that our CFS participants were more inactive than those examined in previous studies. It is also possible that our sedentary control group may not have been as inactive as those used in other studies, even though great care was taken to insure the sedentary nature of the group. While additional accelerometer data on the daily physical activity of sedentary women may exist, comparison of data between studies may prove difficult. Variables such as epoch period, monitor location, monitor type (brand), and calibration may all contribute differences in observed daily physical activity. For that reason, we feel it best to limit our comparison to only those subjects in our own study.

To our knowledge, this study was one of the first to obtain daily ratings of fatigue intensity from a group of CFS participants under normal daily physical activity conditions as well as during periods of increased daily physical activity. Consistent with their diagnosis, CFS patients reported much greater daily ratings of fatigue intensity, time spent each day with fatigue, as well as fatigue recalled during the prior week compared to healthy sedentary participants. This large difference was expected based on the diagnosis and demonstrated the usefulness of ratings of this type to confirm fatigue symptoms.

We found that overall mood, muscle pain intensity, and time spent each day with fatigue worsened following increased activity in our CFS participants compared to controls. Additionally, daily ratings of fatigue intensity (VAS) increased moderately over time while weekly fatigue (POMS) remained stable but elevated in the CFS patients. These observations are contrary to previous studies of increased exercise in this population where exercise training has been shown to reduce fatigue-related symptoms [11,12]. These data demonstrate that, at least in our CFS participants, increases in daily physical activity had no beneficial effects on self-rated fatigue over the measured four week time period.

It is not clear why our study did not find increased daily physical activity to reduce symptom severity, as reported in previous studies [11-13]. One possible explanation is that our fatigue ratings provided a more accurate and discriminatory measure of our participants' fatigue symptoms. By providing them with a 0 to 100 visual analog scale to rate fatigue as well as asking them to assess their symptoms each day we may have obtained a more thorough and accurate picture of their fatigue symptoms than those obtained in previous studies using different rating scales. This is an important distinction between the present study and prior related studies. In our assessment of fatigue, we included daily fatigue intensity and duration. Previous studies have employed measures of fatigue-related symptoms that incorporated more symptoms than simply fatigue intensity or duration. For example, Wearden et al. [12] measured fatigue using the Chalder Fatigue Scale which includes items beyond the scope of fatigue intensity or duration such as sleepiness and "slips of the tongue". Additionally, the reliability of the factor structure of the Chalder Sale has been questioned in the CFS population [14].

Presentation of exercise as a possible treatment for CFS symptoms could have played a role in the improvements in symptom severity observed with exercise training [11-13]. Cognitive behavior therapy has been shown to be beneficial in CFS patients [15], and if CFS participants were made to believe that exercise would be beneficial to them then the observed improvements in other studies could represent some form of a placebo effect. Exact instructions given in previous studies were not reported. Care was taken in this study to present the increase in daily physical activity as a neutral intervention.

A third explanation of our findings is that there may have been something inherent in our exercise protocol that prevented fatigue symptoms from improving. Our method of activity increase, self-paced walking, was no more strenuous than the exercise used in other studies [11-13]. However, the participants were asked to walk every day, as opposed to 2–4 times per week as in other studies, and to attempt to maintain all non-exercise daily activities [11-13]. It is possible the marked increase in activity each day over several weeks had a cumulative effect. By not providing "rest" days, it is possible that the CFS patients were approaching their daily "activity limit." Conversely, while using larger training volumes, previous training studies in this population may not have observed evidence of an "activity limit" due to the fact "rest" days were provided and that 24-hour physical activity levels were not assessed. This hypothesis is based on self-reports that CFS is associated with an inability to sustain normal daily activity levels without a subsequent worsening of symptoms. If the prescribed increase in daily physical activity caused the CFS patients to approach their tolerable activity limit, this could result in a worsening of their fatigue symptoms. Our data suggests that the CFS participants were able to sustain an increase in daily activity over the course of the study. However, it is worth noting that four of our six CFS patients did not reach the goal of a 30% increase in daily activity. Whether this simply represents non compliance is unclear. However, the CFS patients with the lowest baseline daily activity were able to sustain the greatest increase while the patients with the highest baseline activity experienced the smallest increase. This perhaps points toward an activity limit in the CFS patients. An "activity limit' hypothesis might be consistent with the athletic overtraining syndrome in which athletes report heightened feelings of fatigue and loss of energy [16-19]. In addition to fatigue and energy loss, other features of overtraining such as immune dysfunction are similar to those observed in CFS [1,2,20].

Our prescribed exercise was less intense and occurred over a shorter time period than that of previous studies [11-13]. It is possible that more intense exercise over a longer time period is needed to see improvements in fatigue symptoms, and that we simply did not exercise our participants enough to see any improvements. This seems unlikely to us. Although not objectively measured, our CFS participants did subjectively report that maintaining the increased daily activity was difficult for them and they expressed doubts about the possibility of a further increase their daily activity levels.

Five of our six CFS participants also reported having fibromyalgia. Fibromyalgia is a related syndrome with a primary symptom of muscle pain and tenderness [21]. Interestingly, our CFS-FM participants reported no reduction in the intensity of their daily muscle pain as their daily physical activity increased. Similar to their ratings of fatigue intensity, muscle pain showed a trend toward worsening as activity was increased. Exercise programs have been shown to have modest benefits with respect to muscle pain in FM patients [22,23], and it is possible that our muscle pain results should be interpreted in a similar manner to our fatigue results.

It is important to note that our small sample size is a key limitation in our study. The study is under powered to detect differences between our groups. Most of the measured variables had small to moderate effect sizes (Eta^2 ^values from 0.101 to 0.377), suggesting that more participants would be needed to detect differences between the subject groups. Consequently, additional studies will be needed to confirm our results.

In conclusion, this study found that individuals with CFS were able to increase their daily physical activity by approximately 28% for four weeks without serious health complications. At baseline activity our CFS participants exhibited significantly lower daily activity than sedentary controls. This reduced level of daily activity was larger than previously reported values[3,4,6]. Large differences were seen between CFS and controls in several fatigue ratings as well as ratings of muscle pain (consistent with an additional diagnosis of FM). Ratings of overall mood, muscle pain, and time spent each day with fatigue worsened as daily activity increased, and ratings of fatigue intensity did not improve. These findings are in contrast to those of prior exercise studies in this population, which have suggested exercise as a possible clinical treatment for CFS. It is possible that our CFS participants were approaching their daily "activity limit" and this prevented improvements in fatigue symptoms. Future studies are needed to understand the complex interaction between daily physical activity and fatigue symptoms, and to determine if a daily "activity limit" can be quantified in CFS patients.
